# Functional Interaction Between *BRCA1* and DNA Repair in Yeast May Uncover a Role of *RAD50, RAD51, MRE11A*, and *MSH6* Somatic Variants in Cancer Development

**DOI:** 10.3389/fgene.2018.00397

**Published:** 2018-09-19

**Authors:** Luisa Maresca, Samuele Lodovichi, Alessandra Lorenzoni, Tiziana Cervelli, Rossella Monaco, Laura Spugnesi, Mariella Tancredi, Elisabetta Falaschi, Katia Zavaglia, Elisabetta Landucci, Manuela Roncella, Caterina Congregati, Angiolo Gadducci, Antonio Giuseppe Naccarato, Maria Adelaide Caligo, Alvaro Galli

**Affiliations:** ^1^Molecular Genetics Unit, Department of Laboratory Medicine, University Hospital of Pisa, Pisa, Italy; ^2^Yeast Genetics and Genomics, Institute of Clinical Physiology, CNR Pisa, Pisa, Italy; ^3^PhD Program in Clinical and Translational Sciences, University of Pisa, Pisa, Italy; ^4^Medical Oncology Unit, University Hospital of Pisa, Pisa, Italy; ^5^Breast Unit, University Hospital of Pisa, Pisa, Italy; ^6^Department of Clinical and Experimental Medicine, Division of Internal Medicine, University Hospital of Pisa, Pisa, Italy; ^7^Department of Clinical and Experimental Medicine, Division of Gynecology and Obstetrics, University Hospital of Pisa, Pisa, Italy; ^8^Department of Translational Research and New Technologies in Medicine and Surgery, University Hospital of Pisa, Pisa, Italy

**Keywords:** *BRCA1* missense variants, DNA repair genes, yeast based-functional assay, breast and ovarian cancer, somatic variants

## Abstract

In this study, we determined if BRCA1 partners involved in DNA double-strand break (DSB) and mismatch repair (MMR) may contribute to breast and ovarian cancer development. Taking advantage the functional conservation of DNA repair pathways between yeast and human, we expressed several BRCA1 missense variants in DNA repair yeast mutants to identify functional interaction between BRCA1 and DNA repair in BRCA1-induced genome instability. The pathogenic p.C61G, pA1708E, p.M775R, and p.I1766S, and the neutral pS1512I BRCA1 variants increased intra-chromosomal recombination in the DNA-repair proficient strain RSY6. In the *mre11, rad50, rad51*, and *msh6* deletion strains, the BRCA1 variants p.C61G, pA1708E, p.M775R, p.I1766S, and pS1215I did not increase intra-chromosomal recombination suggesting that a functional DNA repair pathway is necessary for BRCA1 variants to determine genome instability. The pathogenic p.C61G and p.I1766S and the neutral p.N132K, p.Y179C, and p.N550H variants induced a significant increase of reversion in the *msh2*Δ strain; the neutral p.Y179C and the pathogenic p.I1766S variant induced gene reversion also, in the *msh6*Δ strain. These results imply a functional interaction between MMR and BRCA1 in modulating genome instability. We also performed a somatic mutational screening of *MSH6, RAD50, MRE11A*, and *RAD51* genes in tumor samples from 34 patients and identified eight pathogenic or predicted pathogenic rare missense variants: four in *MSH6*, one in *RAD50*, one in *MRE11A*, and two in *RAD51*. Although we found no correlation between BRCA1 status and these somatic DNA repair variants, this study suggests that somatic missense variants in DNA repair genes may contribute to breast and ovarian tumor development.

## Introduction

**FIGURE 1:**
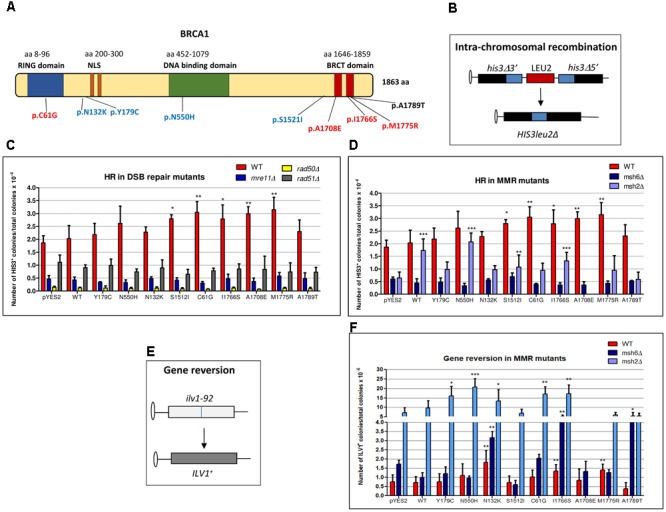
Effect of BRCA1 pathogenic and neutral missense variants on intra-chromosomal recombination and gene reversion in *Saccharomyces cerevisiae*. **(A)** BRCA1 protein is composed by 1863 amino acids (aa) and consists of three main domains (RING, DNA binding, and BRCT domains) and a nuclear localization signal (NLS). Distribution of BRCA1 pathogenic (red), neutral (blue) variants, and VUS (black) tested in *S. cerevisiae.* The number of aa is reported above each functional domain. **(B)** The haploid strain RSY6 carries the two *his3* alleles separated by the *LEU2* marker and by the plasmid DNA sequence, one with a deletion at the 3′ end and the other with a deletion at the 5′ end, which share 400 bp of homology (blue box). An intra-chromosomal recombination event leads to *HIS3* reversion and loss of *LEU2*. **(C)** RSY6 and its derivative DSB repair mutants expressing the *BRCA1*wt or missense variants were inoculated in galactose medium for 17 h at 30°C. As described in Section “Materials and Methods,” cells were counted and plated to score for survival and recombinants. Frequency of intra-chromosomal recombination reported in the vertical axis was expressed as HIS3 colonies per 10^-4^ vital cells. In the horizontal axis, the BRCA1 wt and the variants are reported. Data are reported as mean of four to five independent experiments ± standard deviation. Results were statistically analyzed using the Student’s *t*-test. ^∗^*p* < 0.05, ^∗∗^*p* < 0.01. **(D)** RSY6, *msh2Δ*, and *msh6*Δ mutants expressing the *BRCA1*wt or missense variants were inoculated galactose medium for 17 h at 30°C. Intra-chromosomal HR was determined as described above. In the horizontal axis, the BRCA1 wt and the variants are shown. Data are reported as mean of four to five independent experiments ± standard deviation. Results were statistically analyzed using the Student *t*-test. ^∗^*p* < 0.05, ^∗∗^*p* < 0.01, ^∗∗∗^*p* < 0.001. **(E)** The strain RSY6 carries the *ilv1-92* that allows the assessment of gene reversion to ILV1 by direct counting colonies grown in medium lacking isoleucine. **(F)** RSY6, *msh2Δ*, and *msh6Δ* mutant strains carrying BRCA1 expressing plasmids were inoculated in galactose medium for 17 h at 30°C. As described in Section “Materials and Methods,” cells were counted and plated to score for vital cells and revertants. Frequency of reversion was calculated as total number of ILV1 revertants per 10^-6^ vital cells. In the horizontal axis, the BRCA1 wt and the variants are reported. Data represents the mean of four to five independent experiments ± standard deviation. Results were statistically analyzed using the Student’s *t*-test. ^∗^*p* < 0.05, ^∗∗^*p* < 0.01, ^∗∗∗^*p* < 0.001.

BRCA1 is a tumor suppressor gene that encodes a multi-domain protein of 1863 amino acid involved in a wide array of cellular pathways that maintain genomic stability, including DNA damage-induced cell cycle checkpoint activation, DNA damage repair, protein ubiquitination, higher chromatin hierarchical control as well as transcriptional regulation and apoptosis (Powell and Kachnic, 2003; Narod and Foulkes, 2004; **Figure 1A**). BRCA1 has been shown to localize at DNA double-strand break (DSB) sites and form nuclear foci with RAD51, an essential component of the homologous recombination (HR) (Scully et al., 1997a,b) and with MRE11A–RAD50–NBS1 (MRN complex), a DNA breakage sensor regulating DSB repair through both HR and non-homologous end joining (NHEJ) (Fu et al., 2003; Greenberg et al., 2006). Moreover, BRCA1 is a key component of a protein complex, termed BRCA1-associated genome surveillance complex (BASC) that contains tumor suppressors, DNA damage sensors and signal transducers, including MRN, the mismatch repair (MMR) proteins MLH1, MSH2, and MSH6, the Bloom syndrome helicase BLM, the ATM kinase, DNA replication factor C (RFC), and PCNA. The association of BRCA1 with MSH2 and MSH6 in the BASC complex also links BRCA1 to a sub-pathway of nucleotide excision repair (NER) that repairs base lesions in the transcribed DNA strand (Wang et al., 2000).

Germline *BRCA1* pathogenic variants predispose to an increased lifetime risk of breast and ovarian cancer (Miki et al., 1994). Since the discovery of *BRCA1*, many mutations have been reported and classified which are associated with breast and ovarian cancer and cause the production of truncated and non-functional BRCA1 protein (Walker et al., 2013). In addition, many missense variants, named variants of unknown significance (VUS) have been identified (Carvalho et al., 2007). Their relationship to disease is more difficult to predict since the functional impact is not easily predictable. This may complicate the cancer risk assessment and affect the psychological state of carriers and relatives (Couch et al., 2008). The International Agency for Research on Cancer (IARC) proposed a five-class system-based classification of missense variants: Class 1 (not pathogenic or of no clinical significance), Class 2 (likely not pathogenic or of little clinical significance), Class 3 (uncertain), Class 4 (likely pathogenic), and Class 5 (definitely pathogenic) (Plon et al., 2008; Tavtigian et al., 2008a,b; Lindor et al., 2012; Vallee et al., 2012). Functional assays have been developed to improve BRCA1 VUS classification (Radice et al., 2011; Millot et al., 2012).

Cancer-causing mutations in a tumor suppressor gene such as *BRCA1* are expected to impair the protein’s biological activity. Even if mutations in BRCA1 give the higher susceptibility to develop breast cancer, several data suggest that this predisposition is dependent on a combination of several low penetrance factors (Fanale et al., 2012). Moreover, somatic mutations in DNA repair genes have been recently reported in several cancers including breast cancer (Chae et al., 2016; Nik-Zainal et al., 2016). Although mutations in DNA repair pathways that are functionally related to BRCA1 have been identified (Nik-Zainal et al., 2016), impact of these mutations on BRCA1 activity is very difficult to evaluate.

Although the single-cell model eukaryote *Saccharomyces cerevisiae* has no BRCA1 homolog, main DNA repair pathways are evolutionary conserved (Kowalczykowski, 2015; Groothuizen and Sixma, 2016). Recently, we created a web tool to help the construction of “humanized” yeast strains that could be useful to characterize cancer-associated missense variants (Mercatanti et al., 2017). Therefore, we used yeast as genetic model to investigate the functional interaction between BRCA1 missense variants and DNA repair and to address whether this model system could be useful to evaluate the cancer risk in patients carrying mutations in distinct DNA repair genes.

As the expression of BRCA1 cancer-related variants increased HR in yeast, we previously proposed the “yeast recombination assay” as reliable method to determine the functional impact of VUS (Caligo et al., 2009). Recently, we demonstrated in yeast that MSH2 affects BRCA1-induced HR and, in parallel, we found a high frequency (36%) of *MSH2* somatic mutations in breast and ovarian tumors from BRCA1 missense variant carriers (Maresca et al., 2015). Taking advantage of the functional and sequence homology between human and yeast DNA repair genes (Sung et al., 2000; Liu et al., 2017), we determined whether the expression of several BRCA1 pathogenic and neutral variants in *MSH6, RAD50, MRE11*, and *RAD51* yeast deletion mutants affects HR. Moreover, we studied also the effect of BRCA1 variant expression on gene reversion in both MMR wild-type and defective yeast mutants. This could give some indication to understand the role of DNA repair in BRCA1-driven tumorigenesis.

Finally, to determine if BRCA1 partners involved in HR, NHEJ, and MMR may have a role in breast and ovarian cancer development, we performed a somatic mutational analysis by next generation sequencing (NGS) of *MSH6, RAD50, MRE11A*, and *RAD51* genes in selected breast and/or ovarian tumors from *BRCA1* missense variant carriers, *BRCA1* mutation carriers, and *BRCA1*wt individuals.

## Materials and Methods

### Plasmids, DNA Manipulation

All the plasmids carrying the BRCA1 variants contain the human *BRCA1* cDNA under the yeast galactose inducible promoter *GAL1*p (Westmoreland et al., 2003). Details about the construction of the BRCA1 yeast expression plasmids have been already described (Caligo et al., 2009).

### Yeast Strain

The haploid strain RSY6 of *S. cerevisiae* (a gift from Robert Schiestl, UCLA Los Angeles, CA, United States) has the following genotype: *MAT*a *ura3-52 leu2-3,112 trp5-27 ade 2-40 ilv1-92 arg4-3 his3*Δ*5′-pRS6-his3Δ3′ lys2-801* (Schiestl et al., 1988). RSY6 carries the two *his3* alleles separated by the *LEU2* marker and by the plasmid DNA sequence, one with a deletion at the 3′ end and the other with a deletion at the 5′ end, which share 400 bp of homology. An intrachromosomal recombination event leads to *HIS3* reversion and loss of *LEU2* (Schiestl, 1989; Schiestl et al., 1989; **Figure 1B**). Media preparation and yeast culturing was carried out according to the standard techniques. Yeast was transformed with plasmid DNA by using the litium acetate method with single strand DNA as carrier following the procedure described in Gietz and Schiestl (2007). Transformants were selected in solid medium lacking uracil (SC-URA). Colonies were grown for 4–5 days at 30°C and further analyzed.

### Protein Extract Preparation and Western Blotting

BRCA1 protein level was determined in yeast cell extracts from RSY6 wild-type and DNA repair deletion mutants, transformed with the BRCA1 expression vector after 24-h induction in galactose medium. Single clones were pre-grown in 10–20 ml of SC-URA glucose medium for 24 h at 30°C. Then, cell pellet was washed in water, and split in two aliquots: one was inoculated in 20 ml of SC-URA glucose and the other one in 20 ml of SC-URA galactose. The cultures were incubated at 30°C for 17 h, under constant shaking. Thereafter, pellets were washed twice in ice cold water and re-suspended in 0.5 ml of suspension buffer [50 mM KCl, 5 mM MgCl_2_, 0.1 M EDTA, 25 mM HEPES, 5 mM DTT, 0.3 M (NH_4_)_2_SO_4_, 10% glycerol, pH 7.4] plus 10 μl of protease inhibitor solution (4.4 mg phenylmethylsulfonyl fluoride, 62 mg pepstatin, 50 mg chemostatin, and 725 ml DMSO in 1 ml H_2_O). Total protein extracts were prepared according to the method reported in Maresca et al. (2015). BRCA1p is analyzed using Anti-BRCA1 MoAb Ab4 (clone SD118-Calbiochem). As loading control, we determined the level of the 3-phosphoglycerate kinase (PGK) with the anti-α3PGK antibody from Molecular Probes.

### Yeast Recombination and Reversion Assay

The haploid RSY6 strain of *S. cerevisiae* and its isogenic mutants carrying deletion in *RAD50, RAD51, MRE11, MSH2*, or *MSH6* were used to evaluate the functional interaction between the BRCA1 missense variants and DNA repair. As previously reported, *BRCA1*wt, four pathogenic (pC61G, p1708E, pI1766S, and pM1775R), four neutral (pN132K, pY179C, pN550H, and pS1521I) BRCA1 missense variants, and one VUS (pA1789T) were expressed in yeast by using yeast shuttle vector plasmid carrying the *BRCA1* cDNA under the control of the galactose inducible promoter (Caligo et al., 2009; Maresca et al., 2015; Lodovichi et al., 2016; **Figure 1A**). The effect of BRCA1wt and BRCA1 missense variants on intra-chromosomal HR frequency was evaluated in *rad50Δ, rad51Δ*, and *mre11Δ* mutants following the procedure already reported (Maresca et al., 2015). To determine the frequency of intra-chromosomal recombination, single colonies were inoculated into 5 ml of SC–URA-LEU medium and incubated at 30°C for 24 h. Thereafter, cultures were washed twice in sterile distilled water and counted. For each *BRCA1* variant as well as the *BRCA1*wt and the controls, aliquots containing 10^7^ cells were inoculated in 5 ml of Synthetic Complete – uracil and leucine (SC–URA-LEU) – medium containing 5% galactose. In parallel, the same number of cells was inoculated in 5 ml of SC–URA-LEU glucose containing medium. Both glucose and galactose cultures were incubated at 30°C for 17 h under constant shaking. Thereafter, cells were washed twice counted and appropriate numbers plated onto complete medium to determine the number of vital cells, and onto solid medium lacking histidine. The frequency of intra-chromosomal recombination was calculated as total number of HIS3 colonies per 10^-4^ vital cells. We have also determined the effect of BRCA1 on gene reversion at *ilv1-92* allele (**Figure 1E**) in MMR-proficient RSY6 and in *msh2Δ* and *msh6Δ* mutant strains, by plating yeast-expressing BRCA1wt or BRCA1 missense variants, grown at 30°C for 17 h in 5-ml SC–URA-LEU plus 5% galactose, in medium lacking isoleucine following the standard procedures; the frequency of reversion was calculated as total number of ILV1 revertants per 10^-6^ vital cells (Zimmermann, 1975; Bronzetti et al., 1990b; Galli et al., 1991).

For each BRCA1 variant, a total number of four to six independent experiments were carried out. Results were statistically analyzed using the Student’s *t*-test.

### Patients and Preparation of DNA Samples

For the somatic mutation screening of *MSH6, RAD50, MRE11A*, and *RAD51* genes, 34 patients belonging to hereditary breast and ovarian cancer (HBOC) families were enrolled. This work has been approved by the Ethical Committee for Human Clinical Studies at the University Hospital of Pisa. All patients underwent genetic counseling at the University Hospital of Pisa, Italy. Thirty-one were affected by breast cancer and three by ovarian cancer. Fourteen patients were carriers of *BRCA1* variants (13 missense and one synonymous), eight carried pathogenic mutations in *BRCA1* gene, and 12 were *BRCA1*wt (**Table 1**). *BRCA1* status of the patients is reported in **Table 1**. All patients tested negative for *BRCA2* mutations. Histopathological and clinical features of patients such as tumor histotype, grade, and receptors status are reported in **Supplementary Table S1**.

**Table 1:** *BRCA1* status of patients.

Patient	*BRCA1* status	cDNA change	Protein change	Exon	Classification
P063	Missense variant	c.4484 G>T	p.R1495M	14	1
P258	Missense variant	**c.5365 G>A**	**p.A1789T**	22	VUS
P519	Missense variant	**c.536 A>G; cl648 A>C**	**p.Y179C; p.N550H**	8; 11	1
P534	Missense variant	c.3418 A>G	p.S1140G	11	1
P563	Missense variant	c.2412 G>C	p.Q804H	11	1
P573	Missense variant	c.4956 G>A	p.M1652I	16	1
P614	Missense variant	c.3418 A>G	P.S1140G	11	1
P628	Missense variant	c.4484 G>T	p.D1546N	15	1
P648	Missense variant	c.2521 OT; c.3119 G>A	p.R841W; p.S1040N	11	1
P709	Missense variant	c.3119 G>A	p.S1040N	11	1
P725	Missense variant	c.4956 G>A	p.M1652I	16	1
P881	Missense variant	c.2412 G>C	p.Q804H	11	1
P932	Missense variant	c.3119 G>A	p.S1040N	11	1
P952	Synonymous variant	c.591 C>T	p.C197C	9	1
P1002	WT	–	–	–	–
P1003	WT	–	–	–	–
P1027	WT	–	–	–	–
P1040	WT	–	–	–	–
P1049	WT	–	–	–	–
P1051	WT	–	–	–	–
P1052	WT	–	–	–	–
P1103	WT	–	–	–	–
P1120	WT	–	–	–	–
P1143	WT	–	–	–	–
P1207	WT	–	–	–	–
P1223	WT	–	–	–	–
P39	MUT	c.3598de110	–	11	Pathogenic mutation
P46	MUT	c.3403de1A	–	11	Pathogenic mutation
P122	MUT	c.5492delC	–	24	Pathogenic mutation
P194	MUT	c.5035_5039delCTAAT	–	17	Pathogenic mutation
P325	MUT	c.5266dupC	–	20	Pathogenic mutation
P358	MUT	ex20Δ	–	20	Pathogenic mutation
P439p	MUT	c.5035_5039delCTAAT	–	17	Pathogenic mutation
P485	MUT	c.l380dupA	–	11	Pathogenic mutation

*BRCA1 missense variants are classified according to IARC (International Agency for Research on Cancer) from class 1 benign to class 5 certainly pathogenic as reported in Plon et al. (2008). BRCA1 missense variants tested in yeast deletion strains are reported in bold underlined.*

DNA was extracted from formalin-fixed paraffin-embedded (FFPE) tumor samples after manual microdissection to isolate the tumor area. For each patient, a blood sample was also available.

### Libraries Preparation and NGS Sequencing

*MSH6* (NM_000179.2), *RAD50* (NM_005732.3), *MRE11A* (NM_133487.3), and *RAD51* (NM_005591.3) genes were screened for somatic mutations by NGS on ION Personal Genome machine^TM^ (Thermo Fisher Scientific, Monza, Italy). To amplify the coding sequence and the exon–intron boundaries of the four genes, a custom panel of 163 primer pairs was designed using Ion AmpliSeq^TM^ Designer software^1^. A target region of 12.07 kb was obtained, covering the 94% of the region of interest. Since FFPE DNA is often fragmented, the average length of each amplicon was 100 bp. Libraries preparation and sequencing were performed according to ion PGM protocol (Ion AmpliSeq^TM^ Library Kit 2.0, Ion OT2^TM^ 200 kit, and ion PGM^TM^ Hi-Q^TM^ Sequencing kit; Thermo Fisher Scientific, Monza, Italy; Spugnesi et al., 2016). For the high confidence detection of somatic mutations present at low frequencies in heterogeneous cancer samples and to obtain coverage of 800–1000×, DNA from eight patients were loaded on each 316 v2 chip. The primary data processing was carried out by Torrent suite v5.0.4 and Ion Torrent Variant Caller v5.0. High stringency quality somatic parameters were set: detection of single-nucleotide variants at MAF = 2% and insertions/deletions at MAF = 5%. BAM files were uploaded on Ion Reporter software v5.0^2^ for variant annotation. Variants with *p*-value >0.01 were discarded in order to avoid false positive variant calls. The *p*-value reported by the Ion Reporter software represents the probability that the variant call is incorrect.

Variants fulfilling the following filtering criteria were selected for further analysis:

• Coverage ≥800×
• MAF ≤1% (according to 1000 Genomes Project)
• Present in only one patient.
• Within 20 bp from exon-intron junction.
• VAF (variant allele frequency) ≥3%.

The pathogenicity of each filtered variant was predicted using SIFT, PolyPhen-2, Grantham score, and MutationTaster2 (Grantham, 1974; Kumar et al., 2009; Adzhubei et al., 2010; Schwarz et al., 2014). Moreover, for each filtered variant an extensive literature revision and of the major clinical and genetic databases (dbSNP, ClinVar, LOVD, and COSMIC) was performed, to integrate *in silico* and functional reports for each variant.

According to functional studies in literature and bioinformatics prediction tools, a putative clinical significance was proposed. Variants were classified as follows:

• “Clearly pathogenic” (P): variants already reported in literature and databases as pathogenic.
• “Probably pathogenic” (PP): variants never reported before and predicted pathogenic by two of the three major prediction tools interrogated (SIFT, PolyPhen2, and MutationTaster2).
• “Probably benign” (PB): variants never reported before and predicted benign by two of the three major prediction tools interrogated.
• “Clearly Benign” (B): variants already reported in literature and databases as benign.
• “Unknown”: no sufficient data available from literature, databases, and prediction tools.

Sanger sequencing was performed to confirm the identified variants. For each variant, Sanger Sequencing was also performed in lymphocyte DNA.

## Results

### HR Induced by Pathogenic BRCA1 Variants in Yeast Is Abolished in DNA Repair Deletion Mutants

As the genetic pathways controlling DNA repair and recombination are basically conserved from yeast to humans, we used this microorganism in order to determine the functional relations between BRCA1 and DNA repair. We have previously reported that the expression of pathogenic BRCA1 missense variants increased intra-chromosomal HR both in haploid and diploid yeast strain between two differentially deleted *his3* alleles that share 400 bp homology leading to formation of HIS3wt with loss of LEU2 (**Figure 1B**; Caligo et al., 2009; Maresca et al., 2015). In the present study, we evaluated the effect of the expression of BRCA1wt and several missense variants (**Figure 1A**) on HR frequency, in yeast strains defective for the key players of HR, NHEJ, and MMR to assess which pathways may affect the BRCA1-induced genome instability. Therefore, the following strains were used: the HR defective strains RSY6*rad51Δ*, the NHEJ defective strains RSY6 *mre11Δ* and RSY6*rad50Δ*, and the MMR defective strain RSY6*msh6Δ*. Results from RSY6 and *msh2Δ* strain have been partially published and are reported here for comparison (Maresca et al., 2015).

Previously, we reported that our yeast strains are able to sustain BRCA1 expression driven by a galactose inducible promoter; moreover, in the RSY6*msh2Δ* strain, all the BRCA1 missense variants used in this study are expressed at comparable level (Caligo et al., 2009; Maresca et al., 2015; Lodovichi et al., 2016). Here, we confirmed that each yeast strain expressed the 220 kDa BRCA1 wt protein and the BRCA1 missense variants (**Supplementary Figure S1**). The expression of the pathogenic p.C61G, pA1708E, p.M775R, and p.I1766S, and the neutral pS1512I BRCA1 variant induced a statistically significant increase of intra-chromosomal HR in RSY6 strain as compared to BRCA1wt (**Figure 1C**). The expression of the BRCA1 wt, the neutral variants p.N132K, p.Y179C, and p.N550H, and the VUS p.A1789T did not induce intra-chromosomal recombination in the DNA repair-proficient RSY6 strain (**Figure 1C**). Previously, we reported that the expression of neutral variants p.N132K and p.Y179C induced a statistically significant increase in HR in the diploid yeast strain RS112; this effect is much weaker than the effect induced by the pathogenic variants (Caligo et al., 2009). This may suggest that the haploid strain RSY6 could be more reliable than the diploid strain to discriminate between pathogenic and neutral variants. In general, we confirmed that yeast is a good model to discriminate pathogenic BRCA1 variants from neutral polymorphisms. In the *mre11, rad50*, and *rad51* deletion strains, the expression of BRCA1 variants p.C61G, pA1708E, p.M775R, p.I1766S, and pS1215I did not increase intra-chromosomal HR (**Figure 1C**); this suggests that a functional DNA DSB repair pathway is necessary for BRCA1 pathogenic variants to determine its effect on HR. These results do not allow us to understand the precise mechanism by which BRCA1 pathogenic variants increased HR in yeast. We could speculate that the pathogenic variants could interfere with the “recombination machinery” leading to an accumulation of “intermediate” recombination substrates that could determine a high level of DSB that could be responsible to the HR effect. A similar effect was seen when the human MMR protein MLH1 was expressed in yeast (Shimodaira et al., 1998). Consequently, in the DSB repair deletion strains *mre11, rad50*, and *rad51* no effect on HR was induced by BRCA1 variants indicating that these DNA repair functions are required to BRCA1-induced HR.

In yeast and other eukaryotes, defects in MMR genes also affect HR (Flores-Rozas et al., 2015; Chakraborty and Alani, 2016). Moreover, inactivation of MMR genes such as MSH2 and MSH6 determines an increase of resistance to anticancer drugs that depends on HR (Durant et al., 1999). This confirms a functional interplay between HR and MMR. In the RSY6*msh2* deletion strain, the expression of BRCA1wt and several missense variants increased intra-chromosomal HR with respect to the negative control (pYES2), as already reported (**Figure 1D**; Maresca et al., 2015). On the other hand, in the RSY6*msh6* strain BRCA1 variants had no effect on intra-chromosomal HR indicating that this MMR function is required to BRCA1 variants to determine the effect on HR.

### Expression of BRCA1 Variants Induced Gene Reversion in RSY6 Strain and MMR Mutants

As MMR defects are related to a general increase in gene mutation (Bak et al., 2014), we have determined the effect of BRCA1 expression on yeast gene reversion in MMR-proficient RSY6 and in the MMR defective *msh2* and *msh6* deletion mutants. We have used the *ilv1-92* reversion assay because it is one of the most used reversion tests (**Figure 1E**; Zimmermann, 1975; Bronzetti et al., 1990a; Galli et al., 1991). The expression of the pathogenic p.M1775R, p.I1766S, and neutral p.N132K variants induced a statically significant increase of gene reversion as compared to BRCA1wt in RSY6 strain (**Figure 1F**). The pathogenic p.C61G and p.I1766S and the neutral p.N132K, p.Y179C, and p.N550H variants induced a significant increase of reversion in the RSY6 *msh2* deletion strain (**Figure 1F**); the neutral p.N132K and the pathogenic p.I1766S variant induced ILV1 reversion also in the RSY6*msh6* (**Figure 1F**). Moreover, in the *msh6Δ* mutant, the expression of the VUS p.A1789T increased gene reversion (**Figure 1F**). These results indicate that MMR could affect BRCA1-induced genome instability. We could hypothesize that the expression of the BRCA1 missense variant when MMR is defective affects DNA replication fidelity as previously reported (Powell and Kachnic, 2003).

### Identification of DNA Repair Gene Missense Variants in Tumor Samples

BRCA1-mutated breast cancers have been reported to show genome instability mainly due to the defect in DSB repair (Kwei et al., 2010). Previously, we found a high frequency of MSH2 missense variants in BRCA1 VUS carrier patients suggesting a role of MMR in BRCA1 tumorigenesis (Maresca et al., 2015). We thought to screen tumor samples to address whether other DNA repair genes may be related to BRCA1 defective cancers. In this study, 34 patients from HBOC families were enrolled: 31 affected by breast cancer and three by ovarian cancer. The histopathological and clinical features of all patients are reported in **Supplementary Table S1**. A total of 14 patients carried *BRCA1* variants (13 missense and one synonymous), eight carried *BRCA1* pathogenic mutations, and 12 patients were *BRCA1*wt. The classification of the *BRCA1* missense variants and the type of BRCA1 mutations found in the patients were reported in **Table 1**. Most BRCA1 missense variants are classified not pathogenic (IARC class 1). Therefore, we can determine whether the presence of mutations in genes other than BRCA1 could have a role in HBOC. Mean age at diagnosis was approximately 40 years. Most patients developed ductal infiltrating carcinoma (IDC) of high grade (G3) (**Supplementary Table S1**).

To detect somatic mutations in DNA repair genes in breast and ovarian cancer samples, DNA was extracted from FFPE as described in Section “Materials and Methods.” The coding regions and exon–intron boundaries of the MMR gene *MSH6*, and HR/NHEJ genes *RAD50, MRE11A*, and *RAD51* were screened by NGS.

The somatic mutational screening identified 45 different variants annotated by Ion Reporter 5.0.4. Among the variants, 20 were exonic and 25 were intronic; moreover, 19 variants were unique and 26 were reported in more than one patient. All exonic variants were single-nucleotide variations, in detail missense or synonymous.

The pathogenicity of each filtered variant was predicted using SIFT, PolyPhen-2, Grantham score, and MutationTaster2 prediction tools. For missense variants, the effect on protein structure was also evaluated using the online web server HOPE^3^ which collects structural information on the 3D protein structure, sequence annotations in UniProt, and predictions from DAS servers (Venselaar et al., 2010). The intronic variants were analyzed with Human Splicing Finder^4^, which predicts the effect of mutations on splicing signals (Desmet et al., 2009). Moreover, for each variant, an extensive literature revision and searching in the major clinical and genetic databases (dbSNP, ClinVar, LOVD, and COSMIC) was performed, to integrate *in silico* and functional reports for each variant.

After filtering, 17 variants were obtained. They are listed together with prediction tool scores in **Table 2**. The gene harboring the major number of variants was *MSH6* followed by *RAD50, MRE11A*, and *RAD51*. Filtered variants were confirmed by Sanger Sequencing. Seven variants were confirmed in tumor tissues, while ten variants were not confirmed due to Sanger Sequencing limitations (frequency <10%). The variants confirmed by Sanger Sequencing were as follows: MSH6 p.T305T, MSH6 p.N345Y, MSH6 p.A729A, RAD50 p.D675D, RAD50p.R850C, RAD50c.3165-4 A>T, and MRE11A: c.1098+17 A>G.

**Table 2:** Variants of DNA repair genes identified in this study.

Gene	Nt change	AA change	VAF (%)	Patient	dbSNP	gMAF	tMAF	SIFT	PolyPhen 2	Grantham	Mutation Taster2	CLINVAR	Clinical significance	Origin
MSH6	c.915T>A	p.T305T	35	P122							P		U	GERMLINE
	c.1033A>T	p.N345Y	30	P258				D	PD	143.0	P		PP	SOMATIC
	c.1281C>T	p.Y427Y	4	P1052							DC		U	SOMATIC
	c.2187C>T	p.A729A	27	P534							DC		U	GERMLINE
	c.2522G>A	p.R841K	9	P881				D	D	26.0	DC		PP	SOMATIC
	c.3023C>T	p.T1008I	3	P1143				D	D	89.0	DC		P	SOMATIC
	c.4001G>A	p.R1334Q	3.5	P1002	rs267608122			D	D	43.0	DC	P	P	SOMATIC
RAD50	c.451C>T	p.L151L	4	P1002		0.00812					DC		U	SOMATIC
	c.1185T>A	p.N395K	3	P1143				T	B	94.0	DC		PB	SOMATIC
	c.2025C>T	p.D675D	72	P614	rs34147298	0.016	0.0				P	B	B	GERMLINE
	c.2548C>T	p.R850C	33	P46	rs181961360	0.001	0.0	D	D	180.0	DC	U	PP	SOMATIC
	c.3165-4 A>T	–	71	P258	rs104895050	0.003	0.0					B	B	GERMLINE
	c.3168A>G	p.E1056E	4	P1052							DC		U	SOMATIC
MRE11A	c.1098+17A>G	–	45	P385	rs1805365	0.0539	0.00421					B	B	GERMLINE
	c.1895C>T	p.S632F	3	P1052				D	PD	145.0	P		PP	SOMATIC
RAD51	c.77C>T	p.S26L	4	P1002				D	B	145.0	DC		PP	SOMATIC
	c.784G>A	p.A262T	5	P1052				D	D	58.0	DC		PP	SOMATIC

*Variants with coverage ≥800X, MAF =1%, present in only one patient and within 20 bp from exon-intron junction were selected for further analysis. Nucleotide (Nt) and amino acid (AA) change, VAF (variant allele frequency), *BRCA1* status, minor allele frequency (MAF) according to 1000 genomes (gMAF, global minor allele frequency; tMAF, minor allele frequency in Tuscany), prediction tools scores, putative clinical significance, and variant origin are reported. SIFT: D, damaging; T, tolerated; PolyPhen 2: D, damaging; PD, possibly damaging; B, benign; MutationTaster2: P, polymorphism; DC, disease causing; CLINVAR: P, Pathogenic; B, benign; U, unknown. Clinical significance: P, clearly pathogenic; PP, probably pathogenic; PB, probably benign; B, clearly benign; U, unknown.*

Since variants with *p*-value >0.01 according to Ion Reporter Software were discarded, these variants are less likely to be false positive. All the variants were also assessed in lymphocyte DNA and five variants were germline and two were somatic. The remaining 10 variants were not detected in the germline, so were considered somatic.

The pathogenic or predicted pathogenic variants, along with the BRCA1 status and the effect on the protein structure predicted by HOPE are reported in **Table 3**. In this cohort of breast and ovarian tumors, four rare variants in *MSH6*, one variant in *RAD50*, one variant in *MRE11A*, and two variants in *RAD51* have been identified. The two *MSH6* variants, p.T1008I and p.R1334Q, were already reported as pathogenic (Castiglia et al., 2008; Wu et al., 2014); the other two variants, p.N345Y and p.R841K, are novel and predicted pathogenic. The *RAD50* variant, p.R850C, is scored pathogenic by all the prediction tools interrogated, and is reported in dbSNP (rs181961360) and in ClinVar databases as unknown alteration. The *MRE11A* variant, p.S632F, is predicted pathogenic and is reported in COSMIC (Id: COSM5793819) in a breast cancer case. The *RAD51* variants p.S26L and p.A262T have never been described before. Two out of eight pathogenic or predicted pathogenic somatic variants were in two *BRCA1* variant carriers, five variants were found in three *BRCA1*wt patients, and one variant out was found in a *BRCA1* mutated patient. So, 14% of the *BRCA1* variant carriers, 25% of the *BRCA1*wt, and 12.5% of the *BRCA1* mutated patients carry also a somatic mutation in this subset of DNA repair genes. Considering patients carrying *BRCA1* variants and BRCA1 pathogenic mutations as a unique category, the distribution of somatic missense variants in *MSH6, RAD50, MRE11A*, and *RAD51* seems not to be affected by *BRCA1* status.

**Table 3:** Pathogenic or predicted pathogenic somatic variants.

Gene	Patient	*BRCA1* status	VAF (%)	AA change	Protein domain	HOPE
MSH6	P258	Missense	30	p.N345Y	–	The mutant residue is bigger than the wild-type; this might lead to bumps. The mutation introduces a more hydrophobic residue; this can result in loss of hydrogen bonds and/or disturb correct folding.
	P881	Missense	9	p.R841K	ATP binding mismatched DNA binding	The mutant residue is located in a domain that is important for binding of other molecules. Mutation of the residue might disturb this function. The mutant residue is smaller; this might lead to loss of interactions.
	P1143	WT	3	p.T1008I	ATP binding mismatched DNA binding	The mutated residue is located in a domain that is important for binding of other molecules and in contact with residues in another domain. It is possible that the mutation disturb these contacts. The wild-type and the mutant residues differ in size and hydrophobicity.
	P1002	WT	3.5	p.R1334Q	–	The charge of the wild-type residue will be lost; this can cause loss of interactions with other molecules or residues. The mutant residue is smaller; this might lead to loss of interactions.
RAD50	P46	MUT	33	p.R850C	ATPase	The mutated residue is located in a domain that is important for the main activity of the protein. Mutation of this residue might disturb protein function. The wild-type and mutant residues differ in charge, size, and hydrophobicity.
MRE11A	P1052	WT	3	p.S632F	Exonuclease	The mutated residue is located in a domain that is important for the main activity of the protein. Mutation of the residue might disturb this function. The mutant residue is bigger; this might lead to bumps. The mutation introduces a more hydrophobic residue at this position. This can result in loss of hydrogen bonds and/or disturb correct folding.
RAD51	P1002	WT	4	p.S26L	DNA binding ATPase recombinase	The mutated residue is located in a domain that is important for the main activity of the protein and is located on the surface of the protein. Mutations at this site can disturb interactions with other molecules or with other domains.
	P1052	WT	5	p.A262T	–	The mutated residue is located in a domain that is important for the main activity of the protein. The differences between the wild-type and mutant residue can disturb the core structure of this important domain and thereby affect the catalytic activity.

*Somatic alterations classified as pathogenic or probably pathogenic variants according to the literature and the prediction tools interrogated. The effect of missense variants on protein structure, obtained from HOPE (http://www.cmbi.ru.nl/hope), is also reported. *BRCA1* status: VUS, variants of unknown significance; MUT, pathogenic mutation; WT, wild type.*

## Discussion

Deficiencies in DNA repair are likely to cause chromosomal instability that leads to cell malfunctioning and tumorigenesis. Genetic polymorphisms in DNA repair genes are very common, and several studies have demonstrated a significant association of these polymorphisms with cancer risk (Goode et al., 2002; Hung et al., 2005; Choudhury et al., 2014; Grundy et al., 2016; Das and Ghosh, 2017). Recently, different DNA repair pathways have been proposed to be jointly involved in cancer (Simonelli et al., 2016). Moreover, to evaluate the efficiency of DNA repair in breast tumor samples may be clinically relevant for therapy (Li et al., 2010; Timms et al., 2014). Particularly, defects in MMR genes are associated with a variety of cancers including sporadic breast cancer (Murata et al., 2005). In this study, we aimed to determine if BRCA1 partners involved in HR, NHEJ, and MMR may contribute to breast and ovarian cancer development.

Taking advantage of yeast genetics and considering the high level of functional conservation of DNA repair pathways between yeast and human, we expressed pathogenic and neutral BRCA1 missense variants in DNA repair yeast mutants to identify functional interaction between BRCA1 and DNA repair proteins (Mohammadi et al., 2015; Abugable et al., 2017). Importantly, this study has confirmed yeast as good system to evaluate the functional impact of clinically relevant BRCA1 missense variants because most pathogenic variants increased HR, gene reversion, or other effects (Caligo et al., 2009; Maresca et al., 2015; Thouvenot et al., 2016). Moreover, we demonstrated the DSB repair pathway and MSH6 protein are required so that BRCA1 missense variants could induce intra-chromosomal HR in yeast suggesting the involvement of BRCA1 in yeast DNA repair. Previously, human MMR gene *MLH1* has been reported to potentially interfere with yeast MMR conferring a mutator phenotype (Shimodaira et al., 1998). Similarly, BRCA1 pathogenic variants could interfere with yeast DNA repair pathways leading to the formation of higher level of endogenous DNA damage that can stimulate both intra-chromosomal HR and gene reversion. In addition, it is possible that different variants (pathogenic or neutral) could functionally interact with specific DNA repair genes and have differential effect when the specific function is lacking.

To address whether DNA repair genes may have a role in BRCA1 tumorigenesis, we performed a sequence analysis in tumor samples from HBOC patients carrying BRCA1wt, BRCA1 mutations, or BRCA1 missense variants. We identified somatic variants in the MMR gene *MSH6* and in the DSB repair genes *RAD50, MRE11A*, and *RAD51.* All genes have at least one rare pathogenic or predicted pathogenic variant. We found pathogenic or predicted pathogenic variants in 14% of *BRCA1* missense variant carriers, in 25% of *BRCA1*wt patients, and in 12.5% of *BRCA1* mutation carriers; therefore, the distribution of these DNA repair variants seems not to be affected by *BRCA1* status. Pathogenic or predicted pathogenic variants were identified in ≅15% of patients confirming the potential involvement of these DNA repair genes in the tumorigenesis process. Due to Sanger sequencing limitations, we were not able to confirm 10 somatic variants at low frequency obtained after filtering. These variants are not false positive according to the *p*-value supplied by Ion Reporter Software. To confirm this evidence, we randomly selected one of these variants (*MSH6* p.R841K) and successfully confirmed it by Droplet Digital PCR.

Two pathogenic and two new predicted pathogenic missense variants were found in *MSH6* gene. These variants were identified in patients carrying *BRCA1*wt (patient P1002 and P1143) or missense variant (patient P258, p.A1789T; patient P881, p.Q804H). Interestingly, the expression of the BRCA1 VUS p.A1789T (patient P258) increased gene reversion when *MSH6* is deleted in our yeast assay. Notably, the expression of the BRCA1 neutral variants p.Y179C and p.N550H (patient P519) increased gene reversion in *MSH2* deleted yeast strain; moreover, this patient has been show to carry a *MSH2* exon deletion (Maresca et al., 2015). These results, together with the high frequency of *MSH2* somatic alterations previously observed in a subset of *BRCA1* carriers suggest that MMR might be frequently impaired in breast cancer (Maresca et al., 2015). It is well known that mutations in MMR genes destabilize the genome and can increase cancer susceptibility and progression. Tumors harboring MMR defects are characterized by the accumulation of mutations at microsatellites repeat sequences (Peltomaki, 2001a,b; Hsieh and Yamane, 2008; Haraldsdottir et al., 2016). Probably, defects in the MMR not only could start malignant transformation but might contribute to a “mutator phenotype.” This condition could lead to the accumulation of genomic aberrations and/or mutations resulting in a more aggressive behavior of the tumor. Germline mutations in *MSH6* are reported to be associated to breast cancer, but no extensive study on evaluation of *MSH6* somatic mutations in breast tumor samples is reported (Ollier et al., 2015; Kraus et al., 2017). It has been reported that mutations in genes encoding for MRN, involved primarily in DNA DSB repair, can increase breast cancer risk (Damiola et al., 2014). Notably, the assessment of DSB repair by measuring RAD51 foci in breast tumor samples has been proposed as predictor of chemo-sensitivity (Asakawa et al., 2010). Here, we have identified a total of four pathogenic predicted variants located in *RAD50, MRE11A*, and *RAD51* genes. Importantly, in the patients carrying these somatic variants, *BRCA1* is mutated (patient P46) or wt (patients P1002 and P1052). Actually, tumor samples from patient P1052 carries two distinct variants one in *MRE11A* and one in *RAD51*, suggesting that also DSB repair may contribute to cancer development. Interestingly, all patients with *BRCA1*wt (P1143, P1002, and P1052) carry mutations in almost all genes analyzed; in particular, patient P1143 has both *MSH6* and *RAD50* mutated, patient P1002 has *MSH6, RAD50*, and *MRE11A* mutated and patient 1052 carries mutations in all four genes analyzed.

Importantly, the functional impact of missense variants found in these screening and located in MSH6, RAD50, MRE11A, and RAD51 gene, was assessed using bioinformatics tools, but it is not completely known if they are functionally linked with BRCA1. Novel assays need to be developed in yeast and /or other model systems to understand their effect DNA repair and functional implications on BRCA1 activity. Altogether, this study suggests that somatic missense variants in DNA repair genes, in particular in MMR pathway, may contribute to breast and ovarian tumor development. Anyhow, we did not find any correlation between *BRCA1* status and these somatic DNA repair variants. These missense variants of DNA repair genes belonging to different pathways may also represent new putative therapeutic targets (Majidinia and Yousefi, 2017).

## Conclusion

The evaluation of functional impact of somatic variants on protein function could be important also to identify novel therapeutic targets. Here, we have proposed a novel functional approach based on yeast as genetic system to fast evaluate the functional interaction between DNA repair pathways and BRCA1 status, in order to give a new clue for precision medicine strategies. Therefore, the exploitation of yeast genetics to evaluate which DNA pathway is required to BRCA1 variant to affect genome stability could be relevant for therapy; combining these data with available genetic data could help geneticists in risk management of BRCA1 VUS carriers (Milne and Antoniou, 2016). Straightforwardly, MMR affects gene reversion in yeast expressing BRCA1 mutated or BRCA1 missense variant, and *MSH2* and *MSH6* deleterious mutations were found in patients carrying *BRCA1* variants. This aspect may strengthen yeast as model system to study functional interrelationship between DNA repair and BRCA1.

## Data Availability

The datasets generated for this study can be found in the Catalogue of Somatic Mutations in Cancer (COSP45736).

## Author Contributions

SL, TC, and AL carried out yeast experiments, analyzed data, and performed statistical analysis. LM, LS, MT, AGal, and MC chose the DNA repair genes for somatic mutational screening. LM performed the NGS analysis and variant prioritization. EF, KZ, EL, MR, CC, AGad, and AN discussed data about patients and tumor samples. LM, SL, and AG wrote the manuscript. All authors approved the final version of the manuscript.

## Conflict of Interest Statement

The authors declare that the research was conducted in the absence of any commercial or financial relationships that could be construed as a potential conflict of interest.

## Footnotes

1 https://www.ampliseq.com/

2 https://ionreporter.thermofisher.com

3 http://www.cmbi.ru.nl/hope

4 http://www.umd.be/HSF3/

## Supplementary Material

FIGURE S1 Expression of human BRCA1 wt and missense variants in the RSY6*wt* and in DNA repair mutant strains of *S. cerevisiae*. BRCA1 was detected in the total protein extracts from yeast strains grown in galactose by Western blot analysis with anti-BRCA1 antibody. Extracts from yeast expressing BRCA1 wild-type and missense variants were loaded as indicated on the top of the figure. In blue extract were prepared from yeasts expressing neutral variants, in red form yeast expressing pathogenic variants, in green from the VUS-expressing yeast, and in black from BRCA1wt or negative control (vector pYES2). The level of BRCA1 was determined in all the strains used: RSY6, *mre11Δ, rad50Δ, rad51Δ*, and *msh6Δ.* Western blot analysis in *msh2Δ* strain was already reported (Maresca et al., 2015). Loading control was evaluated by detecting the level of PGK.

TABLE S1 Histopathological and clinical features of patients. Histotype, grade and receptors status of the tumors are reported.

## References

[B1] Abugable A. A. Awwad D. A. Fleifel D. Ali M. M. El-Khamisy S. Elserafy M. (2017). Personalised medicine: genome maintenance lessons learned from studies in yeast as a model organism. *Adv. Exp. Med. Biol.* 1007 157–178. 10.1007/978-3-319-60733-7_9 28840557

[B2] Adzhubei I. A. Schmidt S. Peshkin L. Ramensky V. E. Gerasimova A. Bork P. (2010). A method and server for predicting damaging missense mutations. *Nat. Methods* 7 248–249. 10.1038/nmeth0410-248 20354512 PMC2855889

[B3] Asakawa H. Koizumi H. Koike A. Takahashi M. Wu W. Iwase H. (2010). Prediction of breast cancer sensitivity to neoadjuvant chemotherapy based on status of DNA damage repair proteins. *Breast Cancer Res.* 12:R17. 10.1186/bcr2486bcr2486 20205718 PMC2879561

[B4] Bak S. T. Sakellariou D. Pena-Diaz J. (2014). The dual nature of mismatch repair as antimutator and mutator: for better or for worse. *Front. Genet.* 5:287 10.3389/fgene.2014.00287 PMC4139959 25191341

[B5] Bronzetti G. Galli A. Della Croce C. (1990a). Antimutagenic effects of chlorophyllin. *Basic Life Sci.* 52 463–468. 10.1007/978-1-4615-9561-8_51 2183784

[B6] Bronzetti G. Morichetti E. Della Croce C. Del Carratore R. Giromini L. Galli A. (1990b). Vanadium: genetical and biochemical investigations. *Mutagenesis* 5 293–295. 2200949 10.1093/mutage/5.3.293

[B7] Caligo M. A. Bonatti F. Guidugli L. Aretini P. Galli A. (2009). A yeast recombination assay to characterize human BRCA1 missense variants of unknown pathological significance. *Hum. Mutat.* 30 123–133. 10.1002/humu.20817 18680205

[B8] Carvalho M. A. Marsillac S. M. Karchin R. Manoukian S. Grist S. Swaby R. F. (2007). Determination of cancer risk associated with germ line BRCA1 missense variants by functional analysis. *Cancer Res.* 67 1494–1501. 10.1158/0008-5472.CAN-06-3297 17308087 PMC2936786

[B9] Castiglia D. Bernardini S. Alvino E. Pagani E. De Luca N. Falcinelli S. (2008). Concomitant activation of Wnt pathway and loss of mismatch repair function in human melanoma. *Genes Chromosomes Cancer* 47 614–624. 10.1002/gcc.20567 18384130

[B10] Chae Y. K. Anker J. F. Carneiro B. A. Chandra S. Kaplan J. Kalyan A. (2016). Genomic landscape of DNA repair genes in cancer. *Oncotarget* 7 23312–23321. 10.18632/oncotarget.81968196 27004405 PMC5029628

[B11] Chakraborty U. Alani E. (2016). Understanding how mismatch repair proteins participate in the repair/anti-recombination decision. *FEMS Yeast Res.* 16:fow071. 10.1093/femsyr/fow071 27573382 PMC5976031

[B12] Choudhury J. H. Choudhury B. Kundu S. Ghosh S. K. (2014). Combined effect of tobacco and DNA repair genes polymorphisms of XRCC1 and XRCC2 influence high risk of head and neck squamous cell carcinoma in northeast Indian population. *Med. Oncol.* 31:67. 10.1007/s12032-014-0067-8 24958516

[B13] Couch F. J. Rasmussen L. J. Hofstra R. Monteiro A. N. Greenblatt M. S. de Wind N. (2008). Assessment of functional effects of unclassified genetic variants. *Hum. Mutat.* 29 1314–1326. 10.1002/humu.20899 18951449 PMC2771414

[B14] Damiola F. Pertesi M. Oliver J. Le Calvez-Kelm F. Voegele C. Young E. L. (2014). Rare key functional domain missense substitutions in MRE11A, RAD50, and NBN contribute to breast cancer susceptibility: results from a Breast Cancer Family Registry case-control mutation-screening study. *Breast Cancer Res.* 16:R58. 10.1186/bcr3669bcr3669 24894818 PMC4229874

[B15] Das R. Ghosh S. K. (2017). Genetic variants of the DNA repair genes from Exome Aggregation Consortium (EXAC) database: significance in cancer. *DNA Repair* 52 92–102. 10.1016/j.dnarep.2017.02.013 28259467

[B16] Desmet F. O. Hamroun D. Lalande M. Collod-Beroud G. Claustres M. Beroud C. (2009). Human splicing finder: an online bioinformatics tool to predict splicing signals. *Nucleic Acids Res.* 37:e67. 10.1093/nar/gkp215 19339519 PMC2685110

[B17] Durant S. T. Morris M. M. Illand M. McKay H. J. McCormick C. Hirst G. L. (1999). Dependence on RAD52 and RAD1 for anticancer drug resistance mediated by inactivation of mismatch repair genes. *Curr. Biol.* 9 51–54. 10.1016/S0960-9822(99)80047-5 9889125

[B18] Fanale D. Amodeo V. Corsini L. R. Rizzo S. Bazan V. Russo A. (2012). Breast cancer genome-wide association studies: there is strength in numbers. *Oncogene* 31 2121–2128. 10.1038/onc.2011.408onc2011408 21996731

[B19] Flores-Rozas H. Jaafar L. Xia L. (2015). The role of DNA mismatch repair and recombination in the processing of DNA alkylating damage in living yeast cells. *Adv. Biosci. Biotechnol.* 6 408–418. 10.4236/abb.2015.66040 26900494 PMC4758339

[B20] Fu Y. P. Yu J. C. Cheng T. C. Lou M. A. Hsu G. C. Wu C. Y. (2003). Breast cancer risk associated with genotypic polymorphism of the nonhomologous end-joining genes: a multigenic study on cancer susceptibility. *Cancer Res.* 63 2440–2446. 12750264

[B21] Galli A. Vellosi R. Fiorio R. Della Croce C. Del Carratore R. Morichetti E. (1991). Genotoxicity of vanadium compounds in yeast and cultured mammalian cells. *Teratog. Carcinog. Mutagen.* 11 175–183. 10.1002/tcm.1770110402 1685805

[B22] Gietz R. D. Schiestl R. H. (2007). High-efficiency yeast transformation using the LiAc/SS carrier DNA/PEG method. *Nat. Protoc.* 2 31–34. 10.1038/nprot.2007.13 17401334

[B23] Goode E. L. Ulrich C. M. Potter J. D. (2002). Polymorphisms in DNA repair genes and associations with cancer risk. *Cancer Epidemiol. Biomarkers Prev.* 11 1513–1530. 12496039

[B24] Grantham R. (1974). Amino acid difference formula to help explain protein evolution. *Science* 185 862–864. 10.1126/science.185.4154.862 4843792

[B25] Greenberg R. A. Sobhian B. Pathania S. Cantor S. B. Nakatani Y. Livingston D. M. (2006). Multifactorial contributions to an acute DNA damage response by BRCA1/BARD1-containing complexes. *Genes Dev.* 20 34–46. 10.1101/gad.1381306 16391231 PMC1356099

[B26] Groothuizen F. S. Sixma T. K. (2016). The conserved molecular machinery in DNA mismatch repair enzyme structures. *DNA Repair* 38 14–23. 10.1016/j.dnarep.2015.11.012S1568-7864(15)30053-7 26796427

[B27] Grundy A. Richardson H. Schuetz J. M. Burstyn I. Spinelli J. J. Brooks-Wilson A. (2016). DNA repair variants and breast cancer risk. *Environ. Mol. Mutagen.* 57 269–281. 10.1002/em.22013 27060854

[B28] Haraldsdottir S. Roth R. Pearlman R. Hampel H. Arnold C. A. Frankel W. L. (2016). Mismatch repair deficiency concordance between primary colorectal cancer and corresponding metastasis. *Fam. Cancer* 15 253–260. 10.1007/s10689-015-9856-210.1007/s10689-015-9856-2 26666765

[B29] Hsieh P. Yamane K. (2008). DNA mismatch repair: molecular mechanism, cancer, and ageing. *Mech. Ageing Dev.* 129 391–407. 10.1016/j.mad.2008.02.012S0047-6374(08)00064-X 18406444 PMC2574955

[B30] Hung R. J. Hall J. Brennan P. Boffetta P. (2005). Genetic polymorphisms in the base excision repair pathway and cancer risk: a HuGE review. *Am. J. Epidemiol.* 162 925–942. 10.1093/aje/kwi318 16221808

[B31] Kowalczykowski S. C. (2015). An overview of the molecular mechanisms of recombinational DNA repair. *Cold Spring Harb. Perspect. Biol.* 7:a016410 10.1101/cshperspect.a016410a016410 PMC4632670 26525148

[B32] Kraus C. Hoyer J. Vasileiou G. Wunderle M. Lux M. P. Fasching P. A. (2017). Gene panel sequencing in familial breast/ovarian cancer patients identifies multiple novel mutations also in genes others than BRCA1/2. *Int. J. Cancer* 140 95–102. 10.1002/ijc.30428 27616075

[B33] Kumar A. Majumdar B. Dutta G. Khandalwal A. G B. Mondal S. (2009). The twiddler’s plus syndrome–a case report. *Kardiol. Pol.* 67 1105–1106. 20017076

[B34] Kwei K. A. Kung Y. Salari K. Holcomb I. N. Pollack J. R. (2010). Genomic instability in breast cancer: pathogenesis and clinical implications. *Mol. Oncol.* 4 255–266. 10.1016/j.molonc.2010.04.S1574-7891(10)00023-2 20434415 PMC2904860

[B35] Li S. X. Sjolund A. Harris L. Sweasy J. B. (2010). DNA repair and personalized breast cancer therapy. *Environ. Mol. Mutagen.* 51 897–908. 10.1002/em.20606 20872853 PMC2962983

[B36] Lindor N. M. Guidugli L. Wang X. Vallee M. P. Monteiro A. N. Tavtigian S. (2012). A review of a multifactorial probability-based model for classification of BRCA1 and BRCA2 variants of uncertain significance (VUS). *Hum. Mutat.* 33 8–21. 10.1002/humu.21627 21990134 PMC3242438

[B37] Liu D. Keijzers G. Rasmussen L. J. (2017). DNA mismatch repair and its many roles in eukaryotic cells. *Mutat. Res.* 773 174–187. 10.1016/j.mrrev.2017.07.001 28927527

[B38] Lodovichi S. Vitello M. Cervelli T. Galli A. (2016). Expression of cancer related BRCA1 missense variants decreases MMS-induced recombination in Saccharomyces cerevisiae without altering its nuclear localization. *Cell Cycle* 15 2723–2731. 10.1080/15384101.2016.1215389 27484786 PMC5053555

[B39] Majidinia M. Yousefi B. (2017). DNA repair and damage pathways in breast cancer development and therapy. *DNA Repair* 54 22–29. 10.1016/j.dnarep.2017.03.009 28437752

[B40] Maresca L. Spugnesi L. Lodovichi S. Cozzani C. Naccarato A. G. Tancredi M. (2015). MSH2 role in BRCA1-driven tumorigenesis: a preliminary study in yeast and in human tumors from BRCA1-VUS carriers. *Eur. J. Med. Genet.* 58 531–539. 10.1016/j.ejmg.2015.09.005 26381082

[B41] Mercatanti A. Lodovichi S. Cervelli T. Galli A. (2017). CRIMEtoYHU: a new web tool to develop yeast-based functional assays for characterizing cancer-associated missense variants. *FEMS Yeast Res.* 17:fox078. 10.1093/femsyr/fox0784562592 29069390

[B42] Miki Y. Swensen J. Shattuck-Eidens D. Futreal P. A. Harshman K. Tavtigian S. (1994). A strong candidate for the breast and ovarian cancer susceptibility gene BRCA1. *Science* 266 66–71. 10.1126/science.7545954 7545954

[B43] Millot G. A. Carvalho M. A. Caputo S. M. Vreeswijk M. P. Brown M. A. Webb M. (2012). A guide for functional analysis of BRCA1 variants of uncertain significance. *Hum. Mutat.* 33 1526–1537. 10.1002/humu.22150 22753008 PMC3470782

[B44] Milne R. L. Antoniou A. C. (2016). Modifiers of breast and ovarian cancer risks for BRCA1 and BRCA2 mutation carriers. *Endocr. Relat. Cancer* 23 T69–T84. 10.1530/ERC-16-0277ERC-16-0277 27528622

[B45] Mohammadi S. Saberidokht B. Subramaniam S. Grama A. (2015). Scope and limitations of yeast as a model organism for studying human tissue-specific pathways. *BMC Syst. Biol.* 9:96. 10.1186/s12918-015-0253-0 26714768 PMC4696342

[B46] Murata H. Khattar N. H. Gu L. Li G. M. (2005). Roles of mismatch repair proteins hMSH2 and hMLH1 in the development of sporadic breast cancer. *Cancer Lett.* 223 143–150. 10.1016/j.canlet.2004.09.039 15890247

[B47] Narod S. A. Foulkes W. D. (2004). BRCA1 and BRCA2: 1994 and beyond. *Nat. Rev. Cancer* 4 665–676. 10.1038/nrc1431 15343273

[B48] Nik-Zainal S. Davies H. Staaf J. Ramakrishna M. Glodzik D. Zou X. (2016). Landscape of somatic mutations in 560 breast cancer whole-genome sequences. *Nature* 534 47–54. 10.1038/nature17676nature17676 27135926 PMC4910866

[B49] Ollier M. Radosevic-Robin N. Kwiatkowski F. Ponelle F. Viala S. Privat M. (2015). DNA repair genes implicated in triple negative familial non-BRCA1/2 breast cancer predisposition. *Am. J. Cancer Res.* 5 2113–2126. 26328243 PMC4548324

[B50] Peltomaki P. (2001a). Deficient DNA mismatch repair: a common etiologic factor for colon cancer. *Hum. Mol. Genet.* 10 735–740. 11257106 10.1093/hmg/10.7.735

[B51] Peltomaki P. (2001b). DNA mismatch repair and cancer. *Mutat. Res.* 488 77–85. 10.1016/S1383-5742(00)00058-2 11223406

[B52] Plon S. E. Eccles D. M. Easton D. Foulkes W. D. Genuardi M. Greenblatt M. S. (2008). Sequence variant classification and reporting: recommendations for improving the interpretation of cancer susceptibility genetic test results. *Hum. Mutat.* 29 1282–1291. 10.1002/humu.20880 18951446 PMC3075918

[B53] Powell S. N. Kachnic L. A. (2003). Roles of BRCA1 and BRCA2 in homologous recombination, DNA replication fidelity and the cellular response to ionizing radiation. *Oncogene* 22 5784–5791. 10.1038/sj.onc.12066781206678 12947386

[B54] Radice P. De Summa S. Caleca L. Tommasi S. (2011). Unclassified variants in BRCA genes: guidelines for interpretation. *Ann. Oncol.* 22(Suppl. 1) i18–i23. 10.1093/annonc/mdq661 21285146

[B55] Schiestl R. H. (1989). Nonmutagenic carcinogens induce intrachromosomal recombination in yeast. *Nature* 337 285–288. 10.1038/337285a0 2643057

[B56] Schiestl R. H. Gietz R. D. Mehta R. D. Hastings P. J. (1989). Carcinogens induce intrachromosomal recombination in yeast. *Carcinogenesis* 10 1445–1455. 10.1093/carcin/10.8.1445 2665967

[B57] Schiestl R. H. Igarashi S. Hastings P. J. (1988). Analysis of the mechanism for reversion of a disrupted gene. *Genetics* 119 237–247. 2840335 10.1093/genetics/119.2.237 PMC1203408

[B58] Schwarz J. M. Cooper D. N. Schuelke M. Seelow D. (2014). MutationTaster2: mutation prediction for the deep-sequencing age. *Nat. Methods* 11 361–362. 10.1038/nmeth.2890 24681721

[B59] Scully R. Chen J. Ochs R. L. Keegan K. Hoekstra M. Feunteun J. (1997a). Dynamic changes of BRCA1 subnuclear location and phosphorylation state are initiated by DNA damage. *Cell* 90 425–435. 9267023 10.1016/s0092-8674(00)80503-6

[B60] Scully R. Chen J. Plug A. Xiao Y. Weaver D. Feunteun J. (1997b). Association of BRCA1 with Rad51 in mitotic and meiotic cells. *Cell* 88 265–275. 9008167 10.1016/s0092-8674(00)81847-4

[B61] Shimodaira H. Filosi N. Shibata H. Suzuki T. Radice P. Kanamaru R. (1998). Functional analysis of human MLH1 mutations in Saccharomyces cerevisiae. *Nat. Genet.* 19 384–389. 10.1038/1277 9697702

[B62] Simonelli V. Leuzzi G. Basile G. D’Errico M. Fortini P. Franchitto A. (2016). Crosstalk between mismatch repair and base excision repair in human gastric cancer. *Oncotarget* 8 84827–84840. 10.18632/oncotarget.1018510185 29156686 PMC5689576

[B63] Spugnesi L. Gabriele M. Scarpitta R. Tancredi M. Maresca L. Gambino G. (2016). Germline mutations in DNA repair genes may predict neoadjuvant therapy response in triple negative breast patients. *Genes Chromosomes Cancer* 55 915–924. 10.1002/gcc.22389 27328445

[B64] Sung P. Trujillo K. M. Van Komen S. (2000). Recombination factors of Saccharomyces cerevisiae. *Mutat. Res.* 451 257–275. 10.1016/S0027-5107(00)00054-3 10915877

[B65] Tavtigian S. V. Greenblatt M. S. Goldgar D. E. Boffetta P. (2008a). Assessing pathogenicity: overview of results from the IARC unclassified genetic variants working group. *Hum. Mutat.* 29 1261–1264. 10.1002/humu.20903 18951436 PMC2966307

[B66] Tavtigian S. V. Greenblatt M. S. Lesueur F. Byrnes G. B. (2008b). In silico analysis of missense substitutions using sequence-alignment based methods. *Hum. Mutat.* 29 1327–1336. 10.1002/humu.20892 18951440 PMC3431198

[B67] Thouvenot P. Ben Yamin B. Fourriere L. Lescure A. Boudier T. Del Nery E. (2016). Functional assessment of genetic variants with outcomes adapted to clinical decision-making. *PLoS Genet.* 12:e1006096. 10.1371/journal.pgen.1006096 27272900 PMC4894565

[B68] Timms K. M. Abkevich V. Hughes E. Neff C. Reid J. Morris B. (2014). Association of BRCA1/2 defects with genomic scores predictive of DNA damage repair deficiency among breast cancer subtypes. *Breast Cancer Res.* 16:475. 10.1186/s13058-014-0475-xs13058-014-0475-x 25475740 PMC4308910

[B69] Vallee M. P. Francy T. C. Judkins M. K. Babikyan D. Lesueur F. Gammon A. (2012). Classification of missense substitutions in the BRCA genes: a database dedicated to Ex-UVs. *Hum. Mutat.* 33 22–28. 10.1002/humu.21629 21990165 PMC3478957

[B70] Venselaar H. Te Beek T. A. Kuipers R. K. Hekkelman M. L. Vriend G. (2010). Protein structure analysis of mutations causing inheritable diseases. An e-Science approach with life scientist friendly interfaces. *BMC Bioinformatics* 11:548. 10.1186/1471-2105-11-548 21059217 PMC2992548

[B71] Walker L. C. Whiley P. J. Houdayer C. Hansen T. V. Vega A. Santamarina M. (2013). Evaluation of a 5-tier scheme proposed for classification of sequence variants using bioinformatic and splicing assay data: inter-reviewer variability and promotion of minimum reporting guidelines. *Hum. Mutat.* 34 1424–1431. 10.1002/humu.22388 23893897

[B72] Wang Y. Cortez D. Yazdi P. Neff N. Elledge S. J. Qin J. (2000). BASC, a super complex of BRCA1-associated proteins involved in the recognition and repair of aberrant DNA structures. *Genes Dev.* 14 927–939. 10783165 PMC316544

[B73] Westmoreland T. J. Olson J. A. Saito W. Y. Huper G. Marks J. R. Bennett C. B. (2003). Dhh1 regulates the G1/S-checkpoint following DNA damage or BRCA1 expression in yeast. *J. Surg. Res.* 113 62–73. 10.1016/S0022-4804(03)00155-0 12943812

[B74] Wu G. Diaz A. K. Paugh B. S. Rankin S. L. Ju B. Li Y. (2014). The genomic landscape of diffuse intrinsic pontine glioma and pediatric non-brainstem high-grade glioma. *Nat. Genet.* 46 444–450. 10.1038/ng.2938 24705251 PMC4056452

[B75] Zimmermann F. K. (1975). Procedures used in the induction of mitotic recombination and mutation in the yeast *Saccharomyces cerevisiae*. *Mutat. Res.* 31 71–86. 10.1016/0165-1161(75)90069-2 235086

