# Supplementary figures and images for: Functional Interaction Between BRCA1 and DNA Repair in Yeast May Uncover a Role of RAD50, RAD51, MRE11A, and MSH6 Somatic Variants in Cancer Development
Source: Front Genet. 2018 Sep 19;9:397. doi: 10.3389/fgene.2018.00397 (PMC6156519; doi:10.3389/fgene.2018.00397)

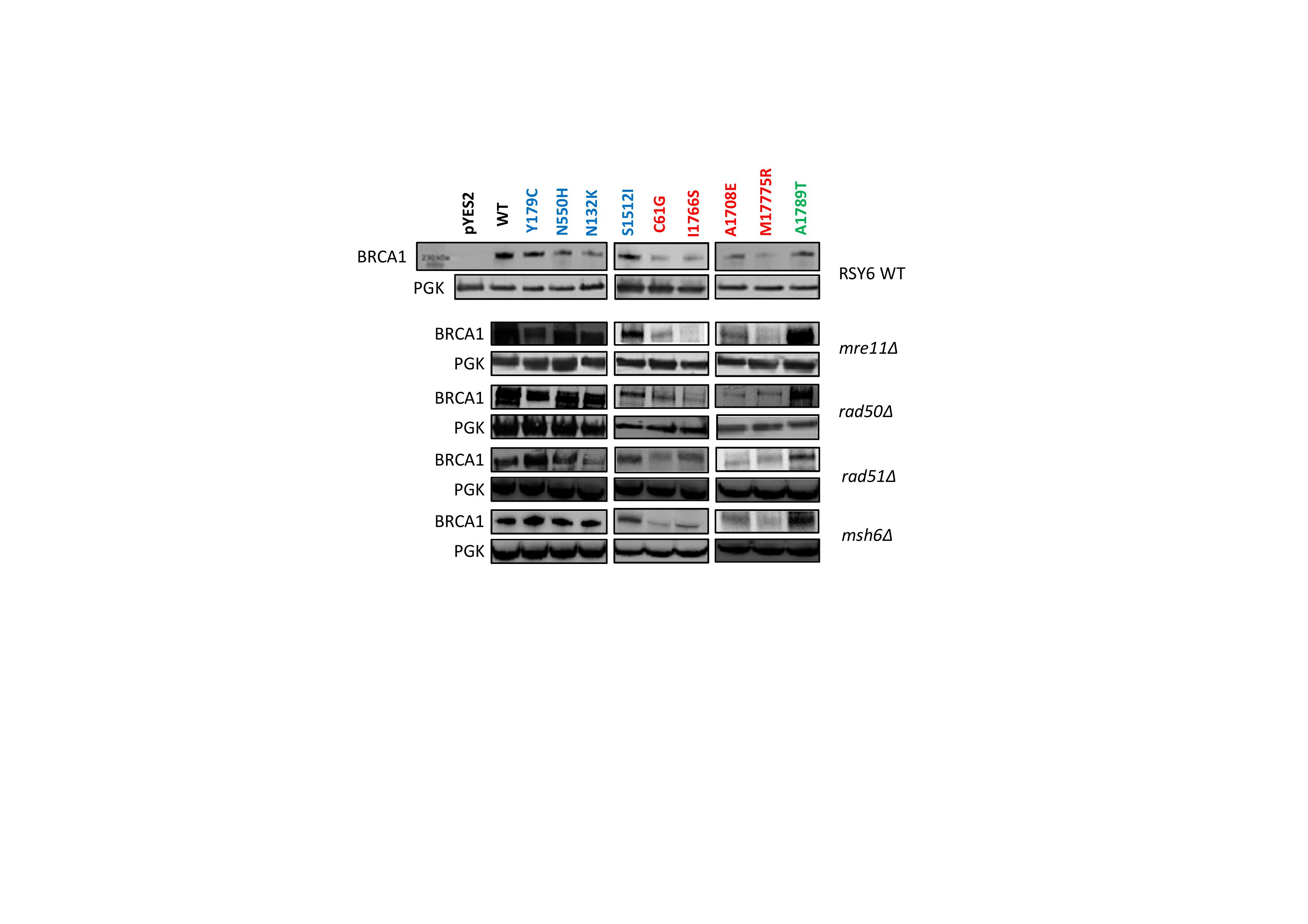

Supplement: FIGURE S1 — Expression of human BRCA1 wt and missense variants in the RSY6wt and in DNA repair mutant strains of S. cerevisiae. BRCA1 was detected in the total protein extracts from yeast strains grown in galactose by Western blot analysis with anti-BRCA1 antibody. Extracts from yeast expressing BRCA1 wild-type and missense variants were loaded as indicated on the top of the figure. In blue extract were prepared from yeasts expressing neutral variants, in red form yeast expressing pathogenic variants, in green from the VUS-expressing yeast, and in black from BRCA1wt or negative control (vector pYES2). The level of BRCA1 was determined in all the strains used: RSY6, mre11Δ, rad50Δ, rad51Δ, and msh6Δ. Western blot analysis in msh2Δ strain was already reported (Maresca et al., 2015). Loading control was evaluated by detecting the level of PGK. [file Image_1.jpg]
