# Supplementary material for: Functional Interaction Between BRCA1 and DNA Repair in Yeast May Uncover a Role of RAD50, RAD51, MRE11A, and MSH6 Somatic Variants in Cancer Development
Source: Front Genet. 2018 Sep 19;9:397. doi: 10.3389/fgene.2018.00397 (PMC6156519; doi:10.3389/fgene.2018.00397)
Supplement: TABLE S1 — Histopathological and clinical features of patients. Histotype, grade and receptors status of the tumors are reported. [file Table_1.DOCX]

**Table S1: Histopathological and clinical features of patients.**

Histotype, grade and receptors status of the tumors are reported.

| **Patient** | **Tumor** | **Istotype** | **Grade** | **Er** | **Pr** | **Her2** | **Age at diagnosis** | ***BRCA1* status** |  |
| --- | --- | --- | --- | --- | --- | --- | --- | --- | --- |
| P063 | BC | IDC | G3 | na | na | na | 44 | missense variant |  |
| P258 | BC | IDC | G3 | na | na | na | 32 | missense variant |  |
| P519 | BC | IDC | G3 | - | - | - | 38 | missense variant |  |
| P534 | BC | IDC | G3 | + | + | - | 46 | missense variant |  |
| P563 | BC | IDC | G3 | + | + | - | 37 | missense variant |  |
| P573 | BC | IDC | G3 | + | + | - | 36 | missense variant |  |
| P614 | BC | IDC | G3 | - | - | + | 30 | missense variant |  |
| P628 | BC bil | DCIS | na | + | + | - | 52 | missense variant |  |
| P648 | BC | IDC | G3 | - | - | + | 23 | missense variant |  |
| P709 | OC | AC | na | na | na | na | 53 | missense variant |  |
| P725 | BC | IDC | G2 | + | + | - | 44 | missense variant |  |
| P881 | BC | IDC | G3 | - | - | + | 40 | missense variant |  |
| P932 | BC | IDC | G2 | + | + | - | 35 | missense variant |  |
| P952 | BC | IDC | G2 | - | - | - | 46 | synonymous variant |  |
| P1002 | BC | IDC | G3 | - | - | + | 69 | WT |  |
| P1003 | BC | DCIS | na | - | - | + | 34 | WT |  |
| P1027 | BC | IDC | G3 | + | + | - | 39 | WT |  |
| P1040 | BC | IDC | G2 | + | + | - | 31 | WT |  |
| P1049 | BC | IDC | G3 | + | + | - | 31 | WT |  |
| P1051 | BC | IDC | G2 | + | + | - | 44 | WT |  |
| P1052 | BC | IDC | G2 | + | + | - | 33 | WT |  |
| P1103 | BC | IDC | G3 | + | + | - | 50 | WT |  |
| P1120 | BC | IDC | G3 | - | - | + | 30 | WT |  |
| P1143 | BC | IDC | G3 | - | - | - | 44 | WT |  |
| P1207 | BC | IDC | G3 | + | + | + | 37 | WT |  |
| P1223 | BC | IDC | G2 | + | + | - | 39 | WT |  |
| P39 | BC | IDC | G3 | na | na | na | 39 | MUT |  |
| P46 | BC | IDC | G3 | - | - | - | 39 | MUT |  |
| P122 | OC | AC | G3 | na | na | na | 39 | MUT |  |
| P194 | OC | na | na | na | na | na | 49 | MUT |  |
| P325 | BC | IDC | G2 | + | - | - | 50 | MUT |  |
| P358 | BC | IDC | G3 | - | - | - | 43 | MUT |  |
| P439 | BC | IDC | G2 | - | - | - | 30 | MUT |  |
| P485 | BC | IDC | G3 | - | - | na | 32 | MUT |  |

Abbreviations: BC: Breast cancer; BCbil: Bilateral breast cancer; OC: ovarian cancer; IDC: Infiltrating Ductal Carcinoma; DCIS: Ductal in situ carcinoma; AC: adenocarcinoma. G2: Intermediate grade (moderately differentiated); G3: High grade (poorly differentiated). Er: Estrogen receptor; Pr: Progesterone receptor; Her2: Human epidermal growth factor receptor 2. na: not available data.
